# Comparative Neuroprotective Effects of *Moringa oleifera* Seed Oil and Aqueous Extract on Cognitive Functions on a High-Fat, High-Fructose Diet Mice: Focus on Senescence Markers

**DOI:** 10.1155/2024/8034401

**Published:** 2024-04-09

**Authors:** Wawaimuli Arozal, Muhamad Sadam Safutra, Agian Jeffilano Barinda, Harri Hardi, Nounik Cheri Dwita, Hee J. Lee

**Affiliations:** ^1^Department of Pharmacology and Therapeutics, Faculty of Medicine, Universitas Indonesia, Jakarta, Indonesia; ^2^Biomedical Sciences, Faculty of Medicine, Universitas Indonesia, Jakarta, Indonesia; ^3^Metabolic Cardiovascular and Aging Cluster, Indonesia Medical Education and Research Institute (IMERI), Faculty of Medicine, Universitas Indonesia, Jakarta, Indonesia; ^4^Clinical Pharmacology Specialist Study Program, Faculty of Medicine, Universitas Indonesia, Jakarta, Indonesia; ^5^Department of Pharmacology, School of Medicine, Kangwon National University, Chuncheon, Republic of Korea

## Abstract

Several studies have demonstrated that *Moringa oleifera* (MO) has different pharmacological properties, including neuroprotective effects. However, the role of MO in preventing brain impairment in high-fat, high-fructose diet (HFFD) remains unknown. This study aimed to investigate the neuroprotective effects of MO leaves aqueous extract (MOE) and moringa seed oil (MOO) against brain impairment in mice with HFFD. Twenty-eight male mice were randomly divided into four groups: normal diet, HFFD, HFFD + MOE 500 mg/kgBW, and HFFD + MOO 2 mL/kgBW. Cognitive function was assessed using the Y-maze and novel object recognition (NOR) tests. The p16, p21, and BDNF expressions were analyzed using the RT-PCR method. Senescence-associated beta-galactosidase (SA-*β*-gal) staining in the brain was also performed. The results showed that administration of MOE or MOO could increase the percentage of alternation and recognition of new objects, prevent the increase of p16 and p21 expression, and ameliorate SA-*β*-Gal activity in the brain. MOO, but not MOE, increased BDNF expression in senescence brains isolated from HFFD mice. The findings indicate that MOO and MOE possess neuroprotective properties, with MOO demonstrating a greater ability to inhibit the brain senescence process compared to MOE.

## 1. Introduction

It was estimated that 13% of the global population was obese in 2015 [1]. According to statistical prediction in the UK, more than half of the population will be clinically obese in 2050 [2]. In obesity, lipids accumulate in subcutaneous and other organs, including the brain [3]. Lipid accumulation tends to secrete proinflammatory cytokines such as TNF-*α*, IL-6, and IL-1, promoting macrophage production of low-grade chronic inflammation. Furthermore, IL-6 and TNF-*α* can block insulin action, leading to systemic insulin resistance [4].

Insulin resistance could exacerbate disruption of the blood-brain barrier (BBB) due to pericyte loss, mainly from excessive glycolysis [5]. In addition, low-grade chronic inflammation by adipose tissue also contributes to BBB disruption through various mechanisms [6]. BBB disruption will draw neuroinflammatory cells into the BBB, resulting in cellular brain senescence [7]. Therefore, obesity and insulin resistance can disrupt BBB and cause cellular brain senescence. Brains with a high number of senescent cells are more susceptible to neurodegenerative diseases, which may impair cognitive function [8].

Brain senescence can be indicated by increased expression of p16, p53, and p21, as well as senescence-associated beta-galactosidase (SA-*β*-gal) [9]. It can also be marked by reduced brain-derived neurotrophic factor (BDNF) expression. Lower expression of BDNF can lead to a reduction in memory performance [10].

*Moringa oleifera* (MO), also known as drumstick tree or Kelor, has been traditionally used in many countries to treat diseases such as paralysis, sores, and skin infections. MO has also been postulated as a potential neuroprotector by various in vivo and in vitro studies of its phytochemical constituents, such as morangin, astragalin, and isoquercitrin. However, MO pharmacological studies regarding its neuroprotective effects are insufficient, with limited in vitro, in vivo, and clinical trial studies [11].

Various parts of the MO plant have different phytochemical compounds traditionally used in herbal medicine. For instance, aqueous extraction (MOE) has a higher flavonoid content, while MO seed oil (MOO) has higher alkaloids, oxalates, and saponin [12]. Different compositions could differ in effectiveness for treating diseases, including their effects on senescence and neurological function. Therefore, we aimed to explore the neuroprotective effect of MOE and MOO in high-fat, high-fructose diet (HFFD) and brain senescence markers.

## 2. Materials and Methods

### 2.1. High-Fat Diet, 25% Fructose, MOO, and MOE Preparation

Research Diets® code D12492 (rodent diet with 60% kcal% fat) was used as a high-fat diet (Research Diets, USA). This research diet comprises 170 g of fat, 197.5 g of carbohydrates, and 203 g of protein per kilogram [13]. Fructose was purchased from Sweet Food Supply Ltd., Bekasi, Indonesia. To produce 25% fructose, 25 g of fructose was diluted with 100 mL of water. MOE was purchased from Javaplant Ltd., Solo, Indonesia. MOE stocks were made by diluting 0.2 g MOE in 4 mL of water, resulting in a 50 mg/mL concentration. MOO was purchased from Kelorina Ltd., Medan, Indonesia.

### 2.2. Drugs and Chemicals

PBS, formalin, distilled water, ketamine, xylazine, beta-actin primer, p16, p21, and BDNF were purchased from Integrated DNA Technologies Ltd., Singapore. MgCl_2_, glutaraldehyde 0.5%, TE buffer, and nuclease-free water were purchased from Biotechnology Grade.

### 2.3. Animal Preparations

The animal research protocol has been approved by our Institutional Ethics Committee (ethical approval number: KET-562/UN2.F1/ETIK/PPM.00.02/2022) and has followed the principle and standard of animal experiments [14]. Ten weeks of DDY strain mice (Biofarma Laboratory, Indonesia) weighing 20–25 g were housed at room temperature of 25°C and 12 hours of light and dark cycles. This twelve-week experimental study used 28 mice divided into four groups of seven each. The first group was fed with a standard diet and water. The second group was fed with HFFD as the positive control. The third group was fed with HFFD and 500 mg/kgBW MOE. The fourth group was fed with HFFD and 2 mL/kgBW MOO. Body weight was measured every week. A high-fat diet and 25% fructose were provided in the animal cage as food and drink. The animal feeding area was supplied with a high-fat diet in experimental and positive control groups, while a 25% fructose solution was added to the animal's water bottle. For twelve weeks, a cage containing three mice was supplemented with 100 mL of fructose and 60 g of a high-fat diet daily. MOO and MOE were given daily to the animal by oral gavage.

### 2.4. Memory Function Test

#### 2.4.1. Novel Object Recognition (NOR) Test

The NOR test is a nonreward animal paradigm based on the spontaneous exploratory behavior of rodents that measures nonspatial working memory. The NOR test involves two sessions, each divided by an intersession interval (ISI). The first session (familiarization trial) allows the animal to explore two similar objects, while the second session (choice trial) replaces one with a novel, unfamiliar one.

The equipment consists of an open field box measuring 40 cm × 40 cm × 40 cm. Each mouse was acclimated to the open field box for six minutes one day before the experiment. The habituation procedure involved transferring the mouse from its home cage and positioning it in the center of an empty, open area. The mice were subsequently transferred to a holding cage [15]. Additionally, we evaluated the mouse for anxiety-like behavior by quantifying the time it spent in the center. For mice with greater anxiety, a 10-minute session may be necessary to meet the minimum exploration criterion [16]. We decided that the minimum exploration time for both objects is 20 s, as suggested [15].

We used 10 minutes for the first session and 20 minutes for ISI. The time spent investigating a new item indicates recognition memory capability; higher is better [17]. Observation with video tracking was performed independently by two investigators (MSS and NCD) who did not know the group allocation. The time spent was calculated using the average measurement of two investigators. NOR test results were displayed as a discrimination index, which is stated in the following equation:

(1) $$\begin{array}{c} Discrimation\;index=\frac{novel\;object\;exploration\;time-familiar\;object\;exploration\;time}{novel\;object\;exploration\;time+familiar\;object\;exploration\;time}. \end{array}$$

#### 2.4.2. Y-Maze Test

The Y-maze assesses short-term memory in mice through spontaneous alternation, a measure of spatial working memory. Intact prefrontal cortical functions enable mice to remember previously visited arms and avoid less recently visited ones [18]. A high percentage alternation is a high proportion of entries into consecutive arms.

We used a light-grey colored polyvinylchloride *Y*-shaped compartment (28 × 6 × 18 cm) with equal-length arms. In the 13th week, mice were placed in a behavioral testing room for one hour before the test to be acclimatized. The timer was initiated after a mouse was inserted into the start arm. The investigator logs the duration of the mouse's exit from the starting arm and the time spent in each arm it subsequently enters. The raw data included the latency to exit the starting arm and the sequence of arm entries [19]. The spontaneous alteration was performed by placing the mouse in the Y-maze for eight minutes. Percentage alternation was calculated by the following equation:

(2) $$\begin{array}{c} \%\;Alternation=\frac{Number\;of\;alternations}{Total\;number\;of\;arm\;entries-2}\times 100. \end{array}$$

### 2.5. Real-Time Quantitative Reverse Transcription Polymerase Chain Reaction (RT-qPCR)

Total RNA was isolated from hippocampus tissue with the Direct-zol RNA Miniprep Kit. NanoDrop™ spectrophotometer (Thermo Fischer Scientific, USA), with an absorbance ratio of 260 nm, was used to measure RNA purity and concentration. The isolated total RNA was converted into complementary DNA (cDNA) using ReverTra Ace® qPCR RT Master Mix with gDNA Remover (Toyobo, Japan) with 500 ng mRNA template concentration. RT-qPCR was performed with SensiFAST™ SYBR® No-NOX (Meridian Bioscience, USA) with the primer sequence as shown in Table 1. A final concentration of 0.8 *µ*M was used to examine *β*-actin (control), p16, and p21 under the following thermal cycling conditions for 40 cycles: 95°C for 2 minutes, 95°C for 5 min, 60°C for 10 min, and 72°C for 20 min. BDNF cycling conditions were 95°C for 2 minutes, 95°C for 5 min, 58°C for 5 min, and 72°C for 20 min. Relative quantification of mRNA was performed using the Livax method.

The senescence markers were investigated by analyzing p^16INK4a^ and p21^WAF1/Cip1^ as the cyclin-dependent kinase inhibitors, the downstream level of retinoblastoma and p53, at the mRNA levels. In addition, brain-derived neurotrophic factor (BDNF), a neurotrophic factor involved in neurogenesis by activating CREB signaling, will also be analyzed at the mRNA level [20]. p21 and p53 proteins are crucial for the early senescence stage, whereas BDNF is associated with cerebral atrophy and cognitive decline [21, 22]. These markers will be performed using real-time PCR (RT-PCR).

### 2.6. Mice Euthanasia

The mice were promptly euthanized by cervical dislocation after the behavior test. Whole brains were harvested and rinsed with ice-cold buffer saline to remove residual blood for the SA-*β*-gal test. Additionally, the hippocampus was collected for RNA extraction.

### 2.7. Senescence-Associated Beta-Galactosidase (SA-*β*-Gal) Test

Senescent cells exhibit enlarged morphology and expanded lysosomal compartment, enhancing the beta-galactosidase activity. *β*-Galactosidase, a lysosomal enzyme, exhibits substantial expression in senescent cells characterized by increased lysosomal activity. Conversely, its expression is relatively low in proliferating cells [23]. Therefore, SA-*β*-gal staining is one of the most used markers to identify senescence [24, 25]. Mice brain tissues were fixated with 0.5% glutaraldehyde, washed with 1 mM MgCl_2_ with pH 6.0, and stained with X-gal (Cell Signaling Technology, Danvers, MA). Overnight incubation was performed at 37°C. Senescence cells will produce *β*-galactosidase enzyme that reacts with X-gal, producing a greenish blue in brain tissue.

### 2.8. Statistical Analysis

Statistical analyses were performed by IBM SPSS Statistic 22. Numerical results are shown as mean ± SEM (standard error of the mean). One-way ANOVA with post hoc Tukey was performed to measure the significance of body weight, Y-maze test, NOR test, p16 expression, p21 expression, and BDNF expression. The *p* values of ^*∗*^*p* < 0.05 and ^*∗∗*^*p* < 0.01 were considered statistically significant. GraphPad Prism version 9.5.0 was used to display all graphs.

## 3. Results and Discussion

Different parts of MO have been studied for cognitive functional tests in mice, such as ethanolic MO seed extract [26] and methanolic MO-leaved extract [27]. Our previous study showed that MOO and MOE also have the potential for a scopolamine-induced memory impairment model [28]. Therefore, we conducted research in MOO and MOE for the HFFD mice model. We utilized the HFFD mice model due to its superiority in impairing memory function. Another study found that the HFFD model increased the time spent in the NOR test more effectively than the high-fat diet model [29].

Our HFFD model effectively elevated body weight and senescence markers while reducing cognitive performance. In agreement with our study, HFFD produced oxidative stress and eventual cellular senescence in the hippocampal region of the mouse brain, including a decrease in glutamate and glutamine [29]. Mice with high-fat diets also have cognitive impairment because of various mechanisms, such as brain insulin resistance, Nrf2 signaling, and amyloid angiopathy [30].

**Figure 1:**
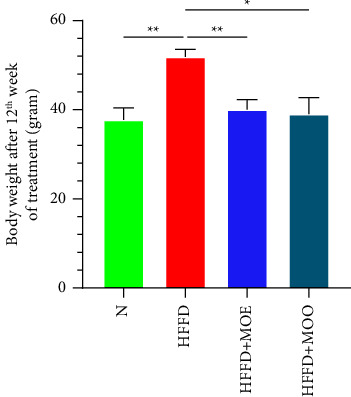
One-way ANOVA analysis of body weight in the 12th week. All data are displayed as mean ± SEM. The *T*-shaped lines on the bars represent the standard error. Normal = normal diet, HFFD = high-fat, high-fructose diet, MOE = aqueous leaves extract of *Moringa oleifera*, MOO = *Moringa oleifera* seed oil. ^*∗*^*p* < 0.05; ^*∗∗*^*p* < 0.01.

There was no difference in body weight between the groups before treatment. Mice fed HFFD tended to gain weight. In contrast, the HFFD + MOE and HFFD + MOO groups exhibited consistent body weights. In the 12th week, our one-way ANOVA analysis revealed statistically significant differences in body weight between the HFFD + MOE and HFFD + MOO groups compared to the HFFD group (Figure 1).

Another study demonstrated that MO could reduce body weight by enhancing glucose tolerance and insulin signaling [31]. MO also increases glucose and lipid metabolism via the AMPK pathway, reducing body weight [32]. Therefore, our study's lower body mass finding in MO groups was consistent with earlier research.

This study only used male mice because previous research has shown that male mice respond better to drugs that affect lifespan. Gender differences in glucose metabolism, drug interactions with sex hormones, and sex-specific drug metabolism contribute to this phenomenon. [33]. Gender differences in human beings have also been attributed to variations in hormones and metabolism. Therefore, it could lead to differences in phenotype and habit in humans, including sex-related drug response [34, 35].

**Figure 2:**
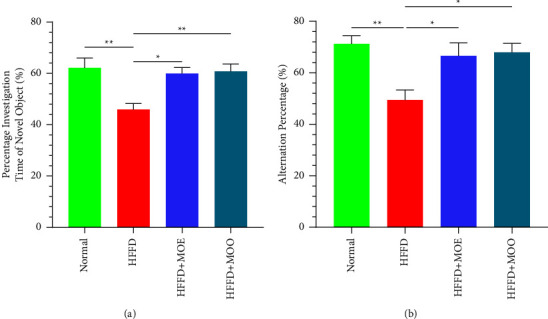
(a) The result of the novel object recognition test showed a percentage investigation time of the novel object; (b) the result of the Y-maze test showed an alternation percentage. All data are displayed as mean ± SEM. The *T*-shaped lines on the bars represent the standard error. Normal = normal diet, HFFD = high-fat, high-fructose diet, MOE = aqueous leaves extract of *Moringa oleifera*, MOO = *Moringa oleifera* seed oil. ^*∗*^*p* < 0.05; ^*∗∗*^*p* < 0.01.

Cognitive function was tested by the NOR test. In our study, the HFFD group spent significantly less time investigating novel objects than the normal group. However, HFFD + MOE and HFFD + MOO groups showed significantly higher percentages in investigation time (Figure 2(a)). Greater significance was observed in the HFFD + MOO group than in the HFFD + MOE group.

The Y-maze test is another alternative to the mice cognitive functional test. Our study revealed that the alternation percentage was lower in the HFFD group. The decline can significantly be ameliorated by supplementing with MOE or MOO. The alternation percentage in the HFFD + MOE and HFFD + MOO groups is more prominent than that in the HFFD group (Figure 2(b)). Based on our NOR and Y-maze tests, MOE and MOO can improve the cognitive performance of HFFD-fed mice. This MO-positive result in NOR and Y-maze tests has been extensively researched in prior studies and is consistent with other research findings [36, 37].

Improvement in cognitive function by MO has been studied and correlated with several mediators such as the Akt-ERK-CREB pathway [38], BACE1 (beta APP-cleaving enzyme 1), and A*β* clearance-associated proteins [27]. To specify that a reduction in senescence can independently cause neurologic improvement, we performed quantitative band analysis in various senescence markers such as BDNF, p16, and p21.

**Figure 3:**
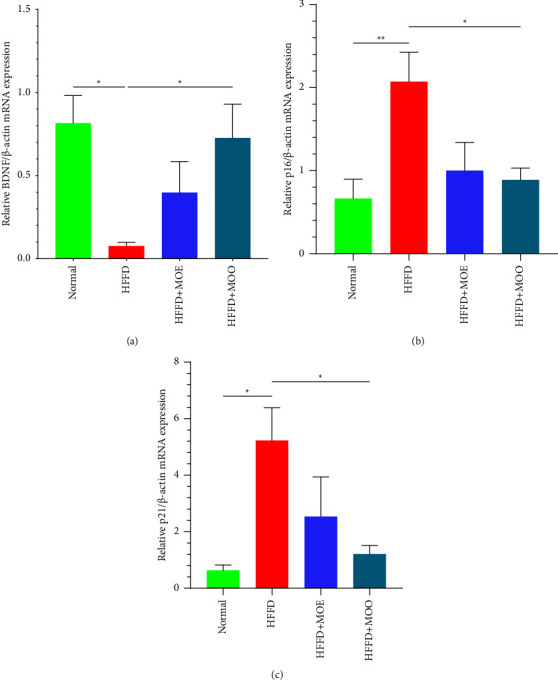
(a) Quantitative band analysis of relative BDNF/*β*-actin mRNA expression; (b) quantitative band analysis of relative p16/*β*-actin mRNA expression; (c) quantitative band analysis of relative p21/*β*-actin mRNA expression. All data are displayed as mean ± SEM. The *T*-shaped lines on the bars represent the standard error. Results that not sharing the same letters in the same graphic are significantly different by ANOVA followed by a Tukey's test (*p* ≤ 0.05). Normal = normal diet, HFFD = high-fat, high-fructose diet, MOE = aqueous leaves extract of *Moringa oleifera*, MOO = *Moringa oleifera* seed oil. ^*∗*^*p* < 0.05; ^*∗∗*^*p* < 0.01.

BDNF is a neurotrophic factor that promotes the survival of senescent neurons and their synapses, preventing cerebral atrophy and cognitive decline [39]. Our study found a reduction in BDNF expression in the HFFD group, which was ameliorated by administering MOO (*p* < 0.05). The HFFD + MOE group also showed an increase relative to the HFFD group, although the difference was not statistically significant (Figure 3(a)). Multiple studies have also established a link between MO supplementation and BDNF-measured cognitive performance [26, 40]. All studies used MOO or methanolic MO leaf extract, revealing a promising result. However, our study showed that MOO was significant, but not with MOE. This result confirms our previous study, which demonstrated that only MOO could increase protein expression levels of TrkB (tropomyosin receptor kinase) and NF-*κ*B, whereas MOE did not [28]. BDNF and TrkB are highly associated with senescence because BDNF has a high affinity for TrkB's primary ligand [41]. However, our previous study showed that MOE can increase the expression of BDNF mRNA in a chronic stress model [42]. This discrepancy could be attributed to the lower dose administered in this study compared with the previous one. The difference between MOO and MOE results may be due to BBB's ability to block the entry of most polar substances, including the polar constituents of MOE, into the central nervous system [43, 44].

As the hallmarks of senescence, p16 and p21 were analyzed in this study. Our study found that p16 and p21 mRNA expression was elevated in the HFFD group, indicating a senescence process. It appears that the senescence process can be mitigated by administering MOO. Compared to the HFFD group, p16 and p21 expression was significantly lower in the HFFD + MOO group. The HFFD + MOE group similarly exhibited a reduction in p16 and p21 expression, but the difference was not statistically significant (Figures 3(b) and 3(c)). Upregulation of p16 and p21 contributes to the acceleration in the senescence process, as it is involved in the aging pathway, p16^INK4A^/pRB, and p53/p21^WAF1/CIP1^ [45]. Another study found that quercetin, one of the flavonoids found in MO, can decrease p16 and p21 expression in murine kidneys fed with a high-fat diet [46]. Quercetin can pass the blood-brain barrier, allowing it to function as a neuroprotector [47]. However, we did not find any study that correlated cognitive function in MO with p16 and p21 expression, as our study did.

**Figure 4:**
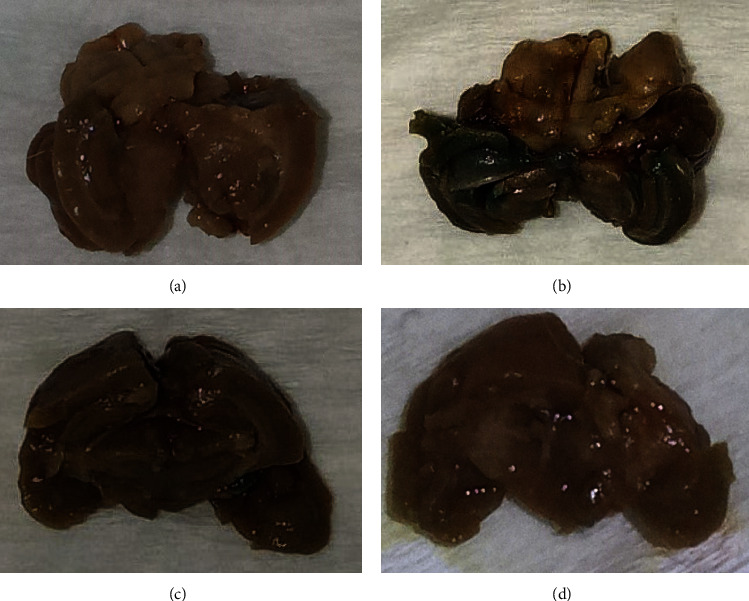
Senescence-associated beta-galactosidase (SA-*β*-gal) test results in brain tissue. Blue stain indicated a positive test result. (a) Mouse with normal diet; (b) mouse with high-fat diet and fructose 25%; (c) mouse with high-fat diet, fructose 25%, and aqueous leaves extract of *Moringa oleifera*; and (d) mouse with high-fat diet, fructose 25%, and *Moringa oleifera* seed oil.

The SA-*β*-gal test only revealed a “greenish blue” color distinction in the HFFD group. This result demonstrated that beta-galactosidase activity increased in the HFFD group (Figure 4). Another study also showed that MO and *Centella asiatica* supplementation could reduce the number of positive SA-*β*-gal-stained cells compared with H_2_O_2_-induced oxidative stress in the human dermal fibroblast model [48]. Another study demonstrated that MOE could decrease the number of senescent cells in lung adenocarcinoma cells [49].

Our study has several limitations. NOR and Y-maze tests can be performed by automated devices to reduce observer bias. SA-*β*-gal can be quantitatively measured using a fluorescence-based assay based on histopathology, such as spider-*β*-gal [50]. We hope that future research will recognize our limitations and encourage more researchers to perform MO studies in senescence.

## 4. Conclusions

Based on NOR and Y-maze tests, MOO (2 mL/kgBW) and MOE (500 mg/kgBW) improved memory impairment in HFFD mice. This outcome could be due to the inhibition of the senescence process, leading to enhancements in several senescence markers, including BDNF, p16, p21, and beta-galactosidase activity, particularly in MOO. The findings demonstrated that MOO and MOE have the capacity to enhance memory impairment. However, MOO demonstrated superior activity in brain senescence markers.

## Figures and Tables

**Table 1:** *β*-Actin, p16, p21, and BDNF primer sequence for RT-PCR.

Gene	Primer	Sequence
*β*-actin	Forward	5′-TAATGTCACGCACGATTTCC-3′
	Reverse	5′-TGTTGTCCCTGTATGCCTCT-3′

p16	Forward	5′-GACGGGCATAGCTTCAGCTCAAGCA-3′
	Reverse	5′-GCCACATGCTAGACACGCTAGCATCGC-3′

p21	Forward	5′-GCCACAGGCACCATGTCCAATCCTGG-3′
	Reverse	5′-GCATCGCAATCACGGCGCAACTGCTC-3′

BDNF	Forward	5′-CTGAGCGTGTGTGACAGTATTA-3′
	Reverse	5′-TGGATACCGGGACTTTCTCT-3′

## Data Availability

The data that support the findings of this study are available from the corresponding author, WA, upon reasonable request.
